# Temporal characteristics of hemodynamic responses during active and passive hand movements in schizophrenia spectrum disorder

**DOI:** 10.1038/s41537-025-00654-6

**Published:** 2025-08-06

**Authors:** Harun A. Rashid, Tilo Kircher, Benjamin Straube

**Affiliations:** 1 Department of Psychiatry and Psychotherapy, University of Marburg, Marburg, Germany; 2 Center for Mind, Brain and Behavior (CMBB), Marburg, Germany

**Keywords:** Schizophrenia, Schizophrenia

## Abstract

In healthy individuals, active hand-movements typically elicit earlier neural processing than passive one, reflected by more positive contrast estimates of the first-order temporal derivative (TD) of hemodynamic response function (HRF) in functional MRI (fMRI) analyses. This temporal advantage might be due to prior movement-awareness and predictive mechanisms that support self-other distinction. However, it is unknown whether impaired predictive mechanisms in Schizophrenia Spectrum Disorder (SSD) influence earlier neural processing. Patients with SSD (*n* = 20) and healthy controls (HC; *n* = 20) performed active and passive hand movements, while detected delays in video feedback of their own or another person’s hand. The recorded fMRI data were analysed applying TD to examine timing and second-order dispersion derivative (DD) to evaluate duration of neural responses. Compared to HC, patients with SSD exhibited delayed BOLD responses during active vs. passive movements in the right caudate nucleus, lobule VIII of right cerebellar hemisphere, left superior temporal gyrus, left postcentral gyrus, left thalamus, and left putamen/insula. Furthermore, during active movement with own hand feedback, patients with SSD showed delayed activation in the bilateral putamen and insula. Delayed insula/putamen responses’ were associated with symptom severity. However, these exploratory findings remain not significant after correction for multiple comparisons and attenuated with Spearman’s-rank correlations. Delayed BOLD responses in patients with SSD, particularly in the right cerebellar lobule VIII, left thalamus, and bilateral insula/putamen may contribute to disturbances in the sense of agency. Altered timing/duration of neural responses reflects new insight underlying deficits in predictive and feedback-monitoring mechanisms in SSD.

## Introduction

The core symptoms of schizophrenia spectrum disorder (SSD) include awareness about action and disturbances in the sense of agency, often reflected in passivity phenomena. Patients with SSD frequently experience uncertainty e.g., whether the willingness to initiate movement planning and execution stems from their own volition. This altered experience of control is thought to emerge from a broader neuro-pathophysiological disturbance, such as prediction of movement-related action consequence^1–9^, sensory-motor feedback integration^10–14^, action-consequences monitoring^10,11,15–20^, self-other distinction in the fine-tune gripping and hand movement^21–26^, and feeling of agency control^11,27–34^. Furthermore, SSD is characterised with psychomotor (movement) abormalities^12,35^, motor-dysfunction with neuro-physiological excitation-inhibition imbalance^36,37^, impaired neural coordination across the brain areas manifested as altered theta, beta, and gamma oscillatory activity in EEG/MEG studies^38–41^. Similarly, functional MRI (fMRI) studies in patients with SSD have reported altered connectivity between the thalamus, pons, and cerebellum^42–45^. These pathophysiological alteration might be reflected in the hand movement-related neural responses’ timing and duration in SSD. While impairments in temporal dynamics of predictive processing might impair the brain’s ability to differentiate self-generated from externally-generated events^46^, evidence from fMRI studies in this domain is –to our knowledge- missing.

In healthy control (HC) subjects, functional MRI (fMRI) study with self-initiated (active) hand movement has shown earlier blood oxygenation level dependent (BOLD) response dynamics compared to externally controlled (passive) hand movement, possibly due to efference copy (EC) based predictive mechanisms^47^. Similarly, an electroencephalography (EEG) study with speech articulation task results suggested that movement related neural processes begin before movement onset^48,49^. Where neural processes may develop corollary discharges (CD), which may include awareness of action-willingness and a general preparatory thought about cognitive-motor related neural preparation. CD subsequently forms motor commands specific to the sensorimotor task, and the copy of these commands from CD is known as EC^48,49^. The EC forms the neuro-computational basis of an internally predicted representation of the tasks and their feedback, potentially guides brain activation patterns; dynamic CD and EC may facilitate the distinction between intended and external input by shaping perception in sensorimotor and interoceptive brain networks^47,48^. In this regard, fMRI studies reported reduced BOLD activation patterns in active compared to externally forced hand movement in patterns with SSD^11,50–55^.

Previously, we found that patients with SSD have shown reduced neural activation and suppression balance during movement preparation, but not execution^56^. Which aligns with reported psychomotor abnormalities, impaired CD-EC, and excitation-inhibition imbalance potentially underlying disrupted predictive mechanisms and ego-disturbances in SSD^35,36,56^. Abnormalities in CD, and EC could thus interrupt temporal dynamics of BOLD responses, e.g., timing and duration across the brain. However, how active and passive hand movements induce earlier or later neural activation patterns in patients with SSD are yet to be explored. Therefore, we applied an approach recently used in healthy subjects showing earlier neural processing of active compared to passive movements^47^, and aimed to investigate whether and how the timing of the activation patterns for hand movement-feedback processing is impaired in patients with SSD. This approach considers the temporal derivative (TD) and the dispersion derivative (DD) of the canonical hemodynamic response function HRF corresponding to the BOLD responses’ timing and duration, respectively. We expected that deficits in action-feedback prediction and monitoring might be reflected in the altered temporal dynamics of BOLD responses, particularly during active hand movement. Especially, we expect reduced differences between active and passive movements in patients with SSD due to a reduced active advantage (i.e., delayed processing in active conditions) in areas such as the supplementary motor area, dorsomedial prefrontal cortex, cerebellum, putamen, and the insula. Especially the insula, which is not only involved in movement preparation^56^, but also in the processing of multimodal sensory signals, and may involve in the external-internal subjective awareness^57^. Underlying temporal disturbances may advance insight into the translational models of agency disruption in psychosis and guide future interventions targeting predictive and sensorimotor mechanisms.

## Materials and methods

### Participants

In this study, fMRI data from 20 HC (20 to 55 years old, not diagnosed with brain injury/neurological disorder that may affect brain metabolism, not a first-degree relative to SSD) and 20 patients with SSD (19 with schizophrenia and one with schizoaffective disorder [SAD], patients were having an attested ICD-10 [International Classification of Diseases] diagnosis) were included. All subjects were right-handed. HC group (15 male; age: 38.4 ± 9.9 years old), patients with SSD (4 female; age: 38.5 ± 8.5 years). Patients with SSD were matched in terms of their age, sex, and attained highest educational degree. Information about the subjects, groups, stimuli, and equipment used in this study have been reported in a previous publications^3,56^, here further detailed information is given in Table 1 and in the materials and methods section. The patients were largely oligosymptomatic during the time of the experiment reflected in the Scale for the assessment of positive symptoms (SAPS)^58^. However, compared to HC, patients with SSD showed a significantly higher SAPS score (12.1 ± 9.8): delusions (5.6 ± 5.1), delusions of reference (1.1 ± 1.2), delusions of being controlled (0.5 ± 0.9), and residual positive symptoms (4.2 ± 5.1)^56^. This study was evaluated and approved by the local committee. Participants provided informed consent before the test measurement and were compensated for their participation.

**Table 1:** Demographic and clinical characteristics.

Characteristics	HC (*n* = 20)		SSD (*n* = 20)	
Sex	15 (male)	5 (female)	16 (male)	4 (female)
Attained degree	15 (LS), 5 (US), 2 (PS)		13 (LS), 4 (US), 3 (PS)	
Antipsychotic medication	None		4 (none), 1 (FGA), 15 (SGA)	
Age (Mean ± SD)	38.4 ± 9.9		38.5 ± 8.5	
EHI score	82.8 ± 29.3		86.9 ± 17.5	
MWT-B score	27.1 ± 5.2		27.8 ± 3.9	
d2 score	440.9 ± 85.6		402.8 ± 103.1^a^	
TMT-A (seconds)	31 ± 17		29 ± 7	
TMT-B (seconds)	64 ± 29		80 ± 31	
TMT-(B-A; seconds)	33.45 ± 24.06		**50.45** ± **28.42**	
TMT-(B/A; seconds)	2.26 ± 0.70		2.76 ± 1.08	
WAIS FS score	9.3 ± 1.7		9.7 ± 1.4	
WAIS BS score	8.2 ± 1.7		7.9 ± 1.6	
SPQ-B: CP score	0.9 ± 1.1		**3.8** ± **1.9**	
SAPS score	1.9 ± 2.4		**12.1** ± **9.8**	
Hallucinations	0.3 ± 0.9		2 ± 4.2	
Delusions	0.4 ± 0.7		**5.6** ± **5.1**	
Delusions of reference	0 ± 0		**1.1** ± **1.2**	
Delusions of being controlled	0 ± 0		**0.5** ± **0.9**	
Residual positive symptoms	1.2 ± 1.2		**4.2** ± **5.1**	
SANS score	1.3 ± 2.0		**13.6** ± **12.2**	
Olanzapine Equivalent	0 ± 0		**13.36** ± **12.06**	

Bold values represent significant differences between HC and SSD patients (*p* < 0.05, uncorrected).

*LS* lower secondary, *US* upper secondary, *PS* post-secondary, *EHI* Edinburgh Handedness Inventory, *FGA* first-generation antipsychotics, *SGA* second-generation antipsychotics, *MWT-B* Mehrfachwahl-Wortschatz-Test (multiple choice vocabulary test), *d2* d2 test of attention, *TMT* Trail Making Test, *WAIS* Wechsler Adult Intelligence Scale, *FS* forward span, *BS* backward span, *SPQ-B* Schizotypal Personality Questionnaire-Brief, *CP* Cognitive-Perceptual subscale, *SAPS* Scale for the Assessment of Positive Symptoms, *SANS* Scale for the Assessment of Negative Symptoms, *HC* healthy control, *SSD* schizophrenia spectrum disorder.

^a^indicates missing data for one participant. Values and scores are the mean ± SD (standard deviation).

### Stimuli and equipment

A custom-made MR-compatible passive movement device (PMD) was used, by gripping its handle hand could be moved from the left (home position) to the right end and back to the left home position through a circular arc (central angle: ~30 degrees; trajectory: about 5.5 cm). Initiating planning and the execution of hand movement could be self-generated (active; 50% trials) or moved by the PMD (passive; 50% trials) connected with an air pressure controller. Inside the MRI, the PMD was placed next to the right thigh to facilitate right-hand movement. Handle position within ~30° (movement trajectory) and direction of the hand movement were recorded via light emitting and detecting optical fibre cables integrated into the PMD.

The participants’ right hand were simultaneously recorded via a high-speed camera (MRC High Speed, MRC Systems GmbH, Heidelberg, Germany; refresh rate: 4 ms) and displayed recorded video onto the screen (refresh rate: 60 Hz). During the experiment in the scanner, these visual feedback from the monitor was shown onto a tilted mirror. In the other 50% of trials, a similarly positioned pre-recorded hand from a person of the opposite sex were displayed, moving following their actual hand movement on the PMD. The video feedback was displayed with 6 different delays (0, 83, 167, 250, 334, 417 ms + internal setup delay of 43 ms) from the actual hand movement. These delays correspond to the screen’s refresh rate (0, 5, 10, 15, 20, and 25 frames at 60 Hz). Custom-written software on a computer (Intel® Core™ i5-4570 CPU, 3.20 GHz, 4 GB RAM, AMD Radeon HD8570D Graphics Card, 32-bit operating system, Windows 7 Professional [Microsoft Corporation, 2009]) was used to control the setup.

### Experimental design

A mixed factorial design with the within-subject factors, movement execution (active and passive), video feedback (own and other hand), as well as a between-subjects factor group (patients with SSD and HC) was used. This results in four movements and feedback conditions: self-active, self-passive, other-active, and other-passive.

In the fMRI session, two runs were conducted, each run consisted 48 trials. Each run began with cues (either “Active” or “Passive”), instructing whether it’s an active or passive blocks with sequential 24 trials in that block. Each trial initiates with a “Ready” cue, followed by a randomized video display of the hand (“self” or “other”) grasping the knob/handle of PMD. In active trials, participants could prepare and move their hand actively during the displayed video. While in passive trials, hand movement was programmed to be automatically initiated by the PMD with a 500 ms (additionally, internal delay of compressor and PMD) delay after the camera onset; to keep the similarity in the movement onset timing between the active and passive hand movement. Here equally and randomly distributed 6 different delays between 0 and 417 ms were applied between video feedback and actual hand movement. Subsequently, a “Delay?” cue appeared to respond to perceived delay in the feedback via button press; left hand’s index (no) or middle finger to say yes. Trials concluded with a black screen of inter-trial interval, randomized between 2000 and 5000 ms.

### Procedure

Participants were familiarised with the task and setup in a preparatory behavioural session. Where participants were sitting upright in front of a computer screen. The preparatory session was followed by an fMRI session with 2 runs (each having 48 trials). Inside the MRI scanner, subjects were lying in a supine position, while the PMD was placed onto the right thigh. Participants were directed to keep gripping the PMD handle with their right hand using the index finger and thumb for the upper part, and the rest fingers for the lower part of the handle. Hand movements consisted of an extension from the left (home) position to the right end and consequently move it back from the right end to the left home position. The video feedback were displayed on the screen, but participants viewed it in a tilted mirror mounted in the head coil, inside the MRI scanner. During the active hand movement, the subjects were asked (similar as trained in the preparatory session) to complete the movement in about 1500 ms in order to make it consistent with the passive condition, where the subjects just held the handle and let the PMD executed the hand movement. At the end of the experiment, all subjects filled out a post experiment questionnaire.

### Functional data acquisition

The MRI data was acquired using a 3 Tesla Magnetom Trio Tim scanner (Siemens, Erlangen, Germany) with a 12-channel head coil. For functional MRI data, a T2*-weighted gradient-echo echoplanar imaging sequence (repetition time [TR]: 1650 ms, echo time [TE]: 25 ms, flip angle: 70°) was procured. In each run 330 volumes were collected in a descending order, each covering 34 axial brain slices (matrix: 64 × 64, field of view [FoV]: 192 × 192 mm, slice thickness: 4 mm, voxel size: 3 × 3 × 4.6 mm [comprising a 0.6 mm gap]). Anatomical images were acquired as T1-weighted MPRAGE sequence (TR: 1900 ms, TE: 2.26 ms, flip angle: 9°) with a matrix of 256 × 256, FoV of 256 × 256 mm, slice thickness of 1 mm, and voxel size of 1 × 1 × 1.5 mm (including a 0.5 mm gap).

### Statistical analysis

**Fig. 1:**
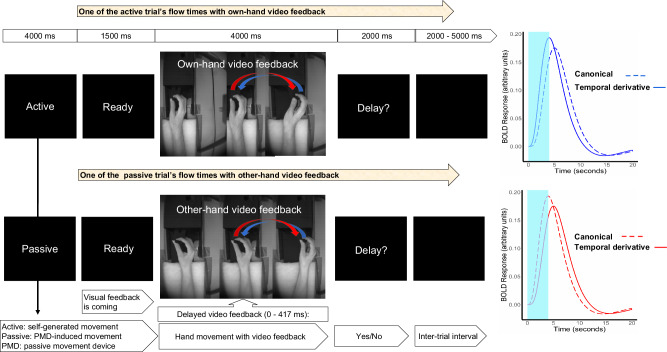
Experimental design. A video demonstration of the hand movement with feedback is available at 10.5281/zenodo.2621302. Each run (48 trials), had two blocks, the block for active movement were beginning with a cue “Active”, and for passive movement with a cue “Passive”. In the beginning of each run participants were instructed to perform hand movement by themselves when cued “Active” (24 trials), or to relax the hand but keep holding the PMD handle when cued “Passive” (24 trials) and let their hand be moved by the PMD. Each trial commenced with a “Ready” cue, followed by visual feedback for movement planning and execution, culminating with a question “Delay?”. The black screen with variable duration (2000 - 5000 ms) is shown during inter trial interval. In the beginning, the hand video feedback was mostly static (1st hand) since the subject was instructed to move when ready to perform the movement. The movement onset from the left (2^nd^ hand) moves to the right (3^rd^ hand), then the hand moves it back to the left (from 3rd hand to the 2nd hand) position. In the video feedback, only the right-hand movement was displayed, here 3 different hand positions are shown just to visualize the whole process of planning and execution direction, separately. Considering male participants for this figure, the upper row shows the sequence of a trial with active and own-hand video feedback, while the lower row shows another trial with the passive and other hand (pre-recorded image from the female). In the case of a female subject, the other hand video is shown from a male hand (pre-recorded image). Self-other hand was displayed randomly across the trial and run. On the right, the expected active and passive movement specific canonical hemodynamic response function and temporal derivative of BOLD responses are shown (the 4000 ms period of hand movement with feedback stimulus marked with light blue).

The standard procedures from Statistical Parametric Mapping (SPM12 version 7, Wellcome Trust Centre for Neuroimaging, University College London, UK) were implemented in MATLAB 2017a (The Mathworks Inc.) to perform statistical analyses of fMRI data. Correlation related analyses were performed using JASP (Jeffreys’s Amazing Statistics Program; version 0.18.3; JASP team, 2024). The preprocessing steps involved: Realignment, Coregistration between anatomical and functional images, Segmentation, Normalization to the Montreal Neurological Institute (MNI) standard space (resampled to a voxel size of 2 × 2 × 2 mm), and smoothing (8 × 8 ×8 mm full-width at half-maximum (FWHM) Gaussian kernel). Considering the best fit for our between-group analyses with recorded voxel size (3 × 3 × 4.6 mm), we choose a Gaussian kernel of 8 mm based on most precious studies’ recommendation: either double^59,60^, or slightly larger than double^61^, or roughly about thrice the voxel size^62,63^. The framewise displacement between consecutive volumes has been computed, only <10% values exceeded 1 mm in any run. Here, the focus is on the fMRI contrast relevant to the complete movement video feedback period (4000 ms; see Fig. 1).

In these analyses in SPM12, the acquired blood oxygenation level dependent (BOLD) response for each trial was modelled from the time of camera onset to camera offset. The preprocessed functional data were implemented in the General Linear Model (GLM). For each participant, different movement conditions and feedback (self-active, self-passive, other-active, and other-passive), cue, and question periods were modelled as the regressor of interest into the GLM. Additionally, head movement was included as control a regressor, and 6-motion parameter were included as a nuisance regressor into the GLM. The GLM with canonical-HRF as basis function analyses typically captures height/amplitude of BOLD response. Which are biased capturing responses similar to its fixed gamma distribution function across the brain, while in reality the neural response (increase/decrease) onset timing, duration, and amplitude (increase/decrease) patterns across the brain and across the individuals are relatively different. It has been reported that assessing the first and second-order multivariate Taylor expansion of the canonical-HRF, such as applying the first-order expansion of canonical-HRF (temporal derivative [TD] with respect to time) could capture small shift in BOLD responses’ onset timing; and second-order expansion (dispersion derivative [DD] with respect to the width) may reflect BOLD responses’ shape or duration^59,64,65^. However, the inclusion of TD alone is insufficient to capture alterations in the shape or duration of the BOLD response, while expanding with DD enables the detection of differences in the duration of neural responses^47,64,66^. It has been described that incorporation of more parameter induces more variation in the parameter estimate, often referred as the bias-variance trade-off^59^, which may also apply to the addition of more basis function to canonical-HRF e.g., TD and DD. It also reported that addition of DD might attenuate the detection sensitivity^67^. Accordingly, we first analysed canonical-HRF + TD to optimally detect timing-related group differences and subsequently conducted a second analysis using HRF + TD + DD to identify duration-related alterations. Standard solutions of SPM12 where used to apply TD and DD into the first level model. The main focus of this study is to capture parameter estimates of TD (positive reflects earlier and negative reflects relatively late response) and DD (positive reflects shorter and negative reflects relatively longer response duration)^47^, subsequently by measuring the contrast between HC and SSD we aim to examine group differences across movement and feedback conditions.

To remove low-frequency noise from the time series a 128 s high pass filter was applied. Finally, we contrasted regressors of interest against the implicit baseline for each participant, and the resulting contrast estimates were entered into a group-level full factorial model. T and F contrast for the TD and DD were created in each participant’s data, for every regressor of movement-feedback condition (self-active, self-passive, other-active, and other passive) in the first level. This is then used in the second-level analyses, yielding 3 contrasts per movement-feedback condition.

These analyses yields cluster based fMRI results, where inferences for spatial extent need to consider acceptable false-positive rates that could be addressed and calculated using Monte Carlo simulation. Which validates whether the reported cluster size indicates a true effect or is merely due to chance (false positive). Monte Carlo simulation involves repeatedly resampling the null data and performing same statistical analyses as was done in the real data. The Monte Carlo simulations were employed with 10,000 iterations resulting a cluster extent threshold of 104 voxels ensuring correction for multiple comparisons at *P* < 0.05, and voxel level threshold of *P* = 0.005, consistent to previous studies^3,56^. This ensures the balance in the often reported Type I and II errors in the fMRI analyses and why such analyses are encouraged^68,69^. Only clusters’ identified by controlling these thresholds are included. Additionally, *p*-values for family wise error (FWE) correction values are provided in Tables 2, 3, and 4. Coordinates are listed using standard MNI152 space. To explore activation timing and duration, we analysed the following T-contrasts within HC and SSD groups and compared them in conjunction analyses, between-group, and interaction contrast: active>passive; contrast for the commonalities: HC (active>passive) $\cap$ SSD (active>passive); contrast for the group differences: HC (active>passive) > SSD (active>passive); interaction with group differences: HC ([Selfact-Selfpas]-[Otheract-Otherpas]) > SSD([Selfact-Selfpas]-[Otheract-Otherpas]). The same analyses were conducted for passive>active contrast. For the condition and group-specific cluster and effect, contrast masking was implemented. To investigate the group-specific effect, HC (active>passive) > SSD (active>passive) masked by HC (active>passive); SSD (active>passive) > HC (active>passive) masked by SSD (active>passive); HC (passive>active) > SSD (passive>active) masked by HC (passive>active); for condition-specific effect, HC([Selfact-Selfpas]-[Otheract-Otherpas])>SSD([Selfact-Selfpas]-[Otheract-Otherpas]) masked by HC (Self-active) were analysed.

**Table 2:** Results regarding activation timing from TD: active > passive.

Cluster label	Cluster extends	X	Y	Z	T	Z_E_	k_E_	P_FWE-coorr._
Common activation timing in HC and SSD: HC (active>passive) ∩ SSD (active>passive)								
Left precentral gyrus	PreCG_L (61.4%), PoCG_L (24.4%), PCL_L (5%), Not assignable (9.1%)	−22	−26	60	5.19	5.07	472	0.189
Right precentral gyrus	PreCG_R (39%), PoCG_R (17.6%), PCL_R (3%), Not assignable (39.9%)	22	−26	58	5.09	4.98	336	0.401
Left supplementary motor area	SMA_L (41.4%), SMA_R (25.2%), MCC_R (18%), MCC_L (14.3%)	6	8	50	4.83	4.74	894	0.020
Right fusiform gyrus	FFG_R (50.3%), CER6_R (30.4%), CERCRU1_R (5.6%)	34	−62	−18	4.43	4.36	611	0.088
Right putamen	PUT_R (23.6%), INS_R (17.6%), IFGorb_R (8.9%), THA_R (12.4%), PAL_R (5.9%), IFGoperc_R (5.4%), Not assignable (21.5%-),	50	20	−4	4.28	4.21	573	0.108
Left cuneus	CUN_L (20.4%), SOG_R (19.4%), SOG_L (14.7%), PCUN_L (13.3%), CUN_R (13.1%), MOG_R (9%), MOG_L (5.6%)	8	−88	28	4.12	4.06	1242	0.004
Left putamen	PUT_L (42.2%), PAL_L (32.3%), Not assignable (22.4%)	−26	−12	0	3.93	3.87	232	0.669
Right middle temporal gyrus	MTG_R (86.4%), ITG_R (6.6%), MOG_R (6.3%)	56	−56	8	3.62	3.58	301	0.482
Right precentral gyrus	PreCG_R (52.8%), IFGoperc_R (34.4%), MFG_R (12.8%)	46	12	36	3.58	3.54	180	0.817
Lobule VI of left cerebellar hemisphere	CER6_L (48.4%), FFG_L (37.7%), CERCRU1_L (13.9%)	−40	−66	−20	3.32	3.28	122	0.944
Right supramarginal gyrus	SMG_R (83.7%), PoCG_R (12.7%)	60	−16	24	3.26	3.23	110	0.961
HC specific activation for self-generated movement in HC: HC (active>passive) > SSD (active>passive) masked by HC (active>passive)								
Right cuneus	CAU_R (46.7%), PUT_R (11%), THA_R (6%), Not assignable (35.7%)	26	8	14	4.51	4.44	700	0.054
Left superior temporal gyrus	STG_L (52.1%), INS_L (32.1%), TPOsup_L (7.3%), ROL_L (5.5%)	−50	0	−2	4.27	4.21	165	0.855
Lobule VIII of right cerebellar hemisphere	CER8_R (32.3%), CER7b_L (19.5%), CER7b_R (12.3%), CER8_L (11.8%), VER8 (6.2%), Not assignable (17.4%)	−2	−76	−44	4.20	4.14	195	0.776
Left postcentral gyrus	PoCG_L (77.1%), PreCG_L (22.9%)	−48	−16	56	3.81	3.76	140	0.912
Left thalamus	THA_L (14.8%), THA_R (9.4%), CAU_L (7.2%), Not assignable (46.5%)	−4	−14	14	4.10	4.04	747	0.042
Left superior temporal gyrus	STG_L (91.4%), MTG_L (%5.9)	−48	−20	6	3.67	3.63	186	0.801
Left putamen	PUT_L (53.7%), INS_L (10.4%), PAL_L (5.7%), Not assignable (27.6%)	−28	−8	14	3.36	3.33	192	0.784
Interaction effect: HC ([Selfact-Selfpas]-[Otheract-Otherpas8]) > SSD ([Selfact-Selfpas]-[Otheract-Otherpas])								
Left insula	INS_L (49.8%), ROL_L (17.8%), PUT_L (9.6%), Not assignable (19.7%)	−38	−4	16	5.03	4.93	669	0.064
Left postcentral gyrus	PoCG_L (79.2%), SMG_L (20.8%)	−62	−10	36	4.21	4.15	226	0.687
Right insula	INS_R (37.8%), IFGoperc_R (9.8%), PUT_R (8.4%), Not assignable (42.1%)	36	12	14	4.10	4.05	225	0.690
Left superior frontal gyrus	SFG_L (50%), MFG_L (49.4%)	−24	36	30	3.90	3.85	172	0.838
HC specific interaction effect for self-generated movement: HC ([Selfact-Selfpas]-[Otheract-Otherpas]) > SSD ([Selfact-Selfpas]-[Otheract-Otherpas]) masked by HC ([Selfact-Selfpas]-[Otheract-Otherpas])								
Left insula	INS_L (47.6%), PUT_L (23.1%), ROL_L (11.2%), Not assignable (23%)	−38	−4	16	5.03	4.93	338	0.397
Right insula	INS_R (40.2%), PUT_R (13.9%), IFGoperc_R (5.1%), Not assignable (39.4%)	36	10	14	3.76	3.72	137	0.918
Specific interaction effect for self-active movement and own hand feedback in HC: HC ([Selfact-Selfpas]-[Otheract-Otherpas]) > SSD ([Selfact-Selfpas]-[Otheract-Otherpas]) masked by HC Self-active								
Left insula	INS_L (61.8%), PUT_L (5%), ROL_L (8.6%), Not assignable (21.8%)	−32	8	2	4.66	4.57	280	0.536
Right insula	INS_R (46%), IFGoperc_R (17.7%), PUT_R (7.3%), Not assignable (27.4%)	36	12	14	4.10	4.05	124	0.941
Interaction effect: SSD([Selfact-Selfpas]-[Otheract-Otherpas]) > HC ([Selfact-Selfpas]-[Otheract-Otherpas])								
Left precentral gyrus	PreCG_L (65.1%), SFG_L (34.9%)	−28	−14	72	4.15	4.09	149	0.893
Left supplementary motor area	SMA_L (53.4%), PCL_L (26.2%), SMA_R (17.7%)	−6	−14	66	4.07	4.02	221	0.701
Left postcentral gyrus	PoCG_L (83%), PreCG_L (10.2%), SPG_L (6.2%)	−40	−34	64	3.84	3.79	176	0.827

Coordinates are listed in MNI space and used AAL 3v1 cluster labelling. Cluster defining threshold: *p* < 0.005, uncorrected, minimum cluster size = 104 voxels (Monte−Carlo cluster level corrected at *p* < 0.05). (Note: FWE-Cluster-corrected values are provided in Table 2); R = right hemisphere; L = left 384 hemisphere.

*CAU* caudate nucleus, *PreCG* precentral gyrus, *PoCG* postcentral gyrus, *SFG* superior frontal gyrus, *MFG* middle frontal gyrus, *SMG* supra-marginal gyrus, *PUT* lenticular nucleus, putamen, *INS* Insula, *PAL* lenticular nucleus, pallidum, *THA* thalamus, *ROL* Rolandic operculum, *SMA* supplementary motor area, *MCC* middle cingulate & paracingulate gyri, *STG* superior temporal gyrus, *MTG* middle temporal gyrus, *ITG* inferior temporal gyrus, *CUN* cuneus, *SOG* superior occipital gyrus, *MOG* middle occipital gyrus, *FFG* fusiform gyrus, *PCUN* precuneus, *SPG* superior parietal gyrus, *TPOsup* temporal pole, superior temporal gyrus, *IFGoperc* inferior frontal gyrus, opercular part, *IFGorb* inferior frontal gyrus, orbitalis, *PCL* paracentral lobule, *CERCRU1* crus I of cerebellar hemisphere, *CER6* lobule VI of cerebellar hemisphere, *CER7b* Lobule VIIB of cerebellar hemisphere, *CER8* lobule VIII of cerebellar hemisphere, *VER8* lobule VIII of vermis.

**Table 3:** Results regarding activation timing from TD: passive > active.

Cluster label	Cluster extend	X	Y	Z	T	Z_E_	k_E_	P_FWE-coorr._
Interaction effect: HC([Selfpas-Selfact]-[Otherpas-Otheract]) > SSD ([Selfpas-Selfact]-[Otherpas-Otheract])								
Left paracentral lobule	PCL_L (12.4%), SMA_R (11.5%), PoCG_L (10.6%), PCUN_L (10.3%), SMA_L (8.4%), SFG_R (8.1%), SPG_R (6.9%), PreCG_R (6.9%), SFG_L (6.7%)	6	−2	76	4.71	4.62	3134	<0.001
Left middle temporal gyrus	MTG_L (50.2%), Not assignable (48.5%)	−48	−32	−6	4.68	4.60	229	0.678
Lobule VIII of left cerebellar hemisphere	CER8_L (59.4%), CER9_L (12.7%), Not assignable (24.2%)	−8	−62	−52	4.25	4.19	244	0.635
Right parahippocampal gyrus	PHG_R (19.3%), HIP_R (19.3%), FFG_R (13.3%)	32	−46	0	3.93	3.88	181	0.814

Coordinates are listed in MNI space and used AAL 3v1 cluster labelling. Cluster defining threshold: *p* < 0.005, uncorrected, minimum cluster size = 104 voxels (Monte–Carlo cluster level corrected at *p* < 0.05). (Note: FWE-Cluster-corrected values are provided in Table 3); R = right hemisphere; L = left hemisphere.

*PCL* paracentral lobule, *SMA* supplementary motor area, *PreCG* precentral gyrus, *PoCG* postcentral gyrus, *PCUN* precuneus, *SFG* superior frontal gyrus, *SPG* superior parietal gyrus, *MTG* middle temporal gyrus, *CER8* lobule VIII of cerebellar hemisphere, *CER9* lobule IX of cerebellar hemisphere, *HIP* hippocampus, *PHG* parahippocampal gyrus, *FFG* fusiform gyrus.

**Table 4:** Results regarding DD: reflected activation duration in active compared to passive movement and vice versa.

Cluster label	Cluster extends	X	Y	Z	T	Z_E_	k_E_	P_FWE-coorr._
Interaction: HC ([Selfact-Selfpas]-[Otheract-Otherpas]) > SSD ([Selfact-Selfpas]-[Otheract-Otherpas])								
Left precentral gyrus	PreCG_L (49.6%), MFG_L (10.7%), SFG_L (7.4%), Not assignable (32.4%)	−40	0	60	5.53	5.44	732	0.038
Right caudate nucleus	CAU_R (21.9%), ACCsup_R (14%), MFG_R (6.2%). IFGoperc_R (5.1%), Not assignable (37%)	32	14	32	4.23	4.19	1812	<0.001
Left superior parietal gyrus	SPG_L (63.8%), MOG_L (10.5%), PCUN_L (9.5%), SOG_L (7.2%), Not assignable (5.6%)	−22	−60	40	3.89	3.86	497	0.146
Right middle temporal gyrus	MTG_R (58.3%), STG_R (25.1%), Not assignable (16.6%)	52	−50	10	3.78	3.75	175	0.818
Right precuneus	PCUN_R (37.3%), SOG_R (34.7%), CUN_R (5.4%), Notnassignable (16.4%)	22	−64	38	3.73	3.70	426	0.222
Right parahippocampal gyrus	PHG_R (52.9%), FFG_R (25.2%), HIP_R (21.8%)	28	−24	−22	3.67	3.64	119	0.946
Right middle temporal gyrus	MTG_R (79%), MOG_R (21%)	48	−72	6	3.53	3.50	143	0.899
Left middle frontal gyrus	MFG_L (79.6%), OFClat_L (7.6%), SFG_L (6.8%)	−40	48	−10	3.49	3.47	132	0.922
Interaction: HC ([Selfact-Selfpas]-[Otheract-Otherpas]) > SSD ([Selfact-Selfpas]-[Otheract-Otherpas]), masked by HC ([Selfact-Selfpas]-[Otheract-Otherpas])								
Left precentral gyrus	PreCG_L (77.1%), MFG_L (22.9%)	−40	0	60	5.53	5.54	140	0.906
Right caudate nucleus	CAU_R (46.6%), ACCsup_R (9.4%), MCC_R (6.4%), Not assignable (35.4%)	16	20	28	4.16	4.12	425	0.223
Lobule VI of left cerebellar hemisphere	CER6_L (57.7%), CERCRU1_L (32.2%), FFG_L (9%)	−26	−72	−24	4.00	3.96	267	0.546
Left superior parietal gyrus	SPG_L (90.2%), PCUN_L (9.8%)	−16	−66	52	3.52	3.49	184	0.792
Group differences HC(passive-active) > SSD(passive-active)								
Left middle occipital gyrus	MOG_L (65.9%), SOG_L (15.3%), SPG_L (10.1%), IPG_L (8.7%)	−28	−80	38	4.07	4.03	346	0.354

Coordinates are listed in MNI space and used AAL 3v1 cluster labelling. Cluster defining threshold: *p* < 0.005, uncorrected, minimum cluster size = 104 voxels (Monte-Carlo cluster level corrected at *p* < 0.05). (Note: FWE-Cluster-corrected values are provided in Table 4); R = right hemisphere; L = left hemisphere.

*CAU* Caudate nucleus, *CUN* cuneus, *PCUN* precuneus, *PreCG* precentral gyrus, *ACCsup* anterior cingulate cortex, supracallosal, *MCC* middle cingulate cortex, *FFG* fusiform gyrus, *MOG* middle occipital gyrus, *SOG* superior occipital gyrus, *SFG* superior frontal gyrus, *MFG* middle frontal gyrus, *IFGoperc* inferior frontal gyrus, opercular part, *OFClat* lateral orbital gyrus, *STG* superior temporal gyrus, *MTG* middle temporal gyrus, *SPG* superior parietal gyrus, *IPG* inferior parietal gyrus, excluding supramarginal and angular gyri, *PHG* parahippocampal gyrus, *HIP* hippocampus, *CER6* lobule VI of cerebellar hemisphere, *CERCRU1* crus I of cerebellar hemisphere

The name of the brain area is defined as the cluster name which has the highest percent contribution to a cluster(using AAL 3v1 cluster labelling)^70^. The cluster extension of brain regions (>5% contribution) with their percentage of contribution are listed in Tables 2, 3, and 4. Only clusters were listed from which more than 50% percent voxel could be labelled. A cluster label with an MNI152 space coordinate (X Y Z) is used to interpret the result. Thus, all of the results are shown here in the MNI152 space’s coordinate. Latency functions (LF) relative to the canonical-HRF of BOLD responses were assessed using methods described in the previous studies^47,65^.

### Exploratory correlation analyses

Here, we investigated how BOLD responses’ timings and durations reflected by eigenvariates of the clusters in SSD patients are associated with negative and positive symptoms. The positive symptoms were scored by subscales of hallucinations (items 1–7), delusions of reference (item 14), delusions of being controlled (item 15)), and residual positive symptoms (items 21–35) of the assessment of positive symptoms (SAPS)^58^. The negative symptoms were assessed using related subscales of affective flattening or blunting (items 1–8), alogia (items 9–13), avolition/apathy (items 14–17), anhedonia/asociality (items 18–22), and attention (items 23–25 of the scale for the assessment of negative symptoms (SANS)^71^. The correlation of positive and negative symptom scores with neural activations timings (extracted eigenvariates) in active movement (bilateral insula/putamen) with own hand video feedback were performed. To explore specificity regarding the positive symptoms, partial correlations were performed by partialling out total SANS score. Analyses were performed using JASP (Jeffreys’s Amazing Statistics Program; version 0.18.3; JASP team, 2024).

## Results

### fMRI results

#### Group commonalities in the temporal dynamics of BOLD responses (active>passive)

**Fig. 2:**
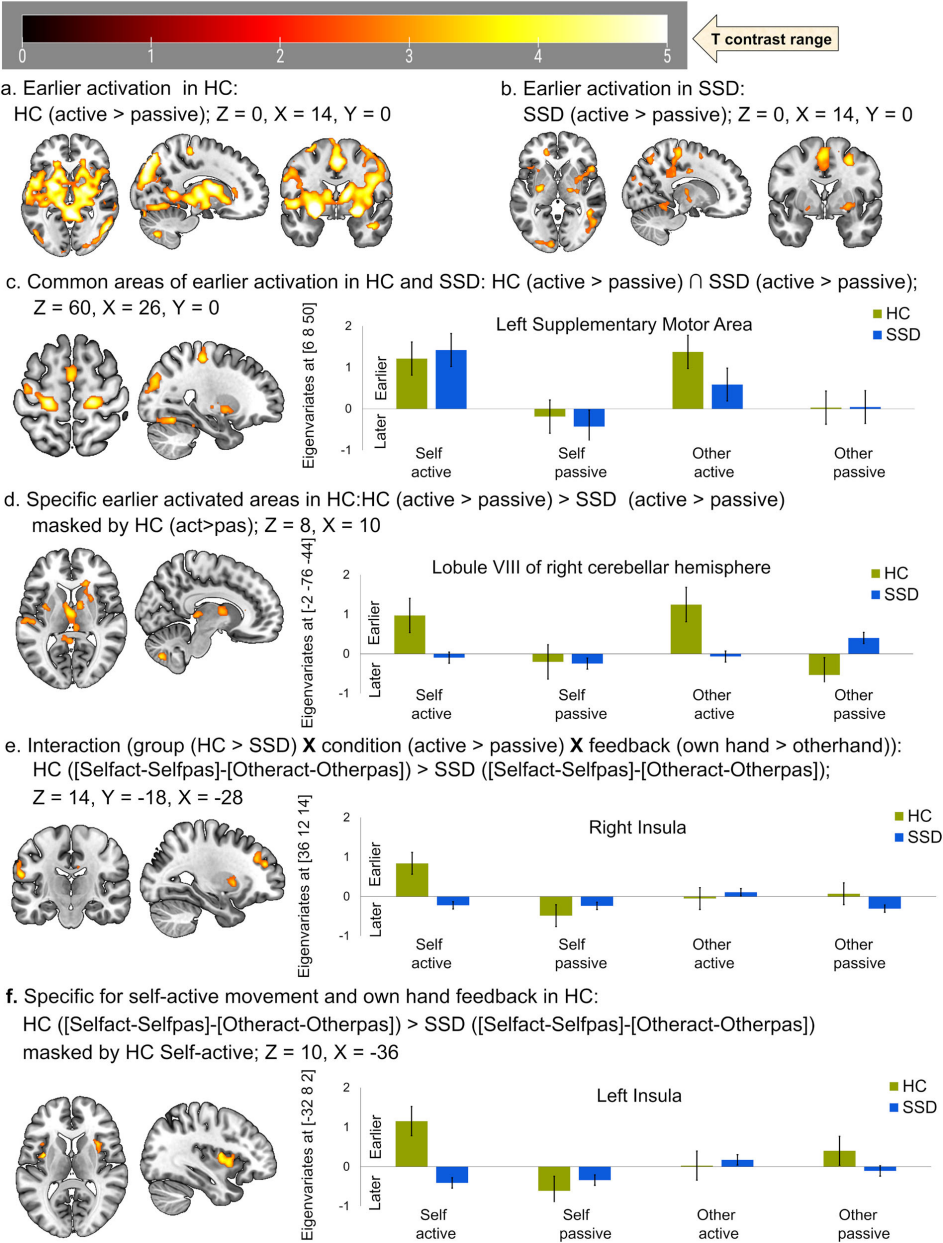
Timing of neural activation in active compared to passive condition. **a** Earlier activation in healthy control (HC) shown at Z = 0, Y = 0, X = 14; (**b**) earlier activation in schizophrenia spectrum disorder (SSD) shown at Z = 0, Y = 0, X = 14; (**c**). common earlier activated brain areas between HC and SSD at Z = 60, Y = 0, X = 26, (**d**) earlier activation specific to HC compared to SSD shown at Z = 8, X = 10; (**e**) interaction related earlier activation in HC compared SSD patients shown at Z = 14, Y = −18, X = −28; **f** active with own hand feedback specific earlier activated brain area in HC compared to SSD patients shown at Z = 10, X = −36. Positive eigenvariates reflect earlier activation while negative reflects later activation timing in the bar graph. HC healthy control, (*n* = 20); SSD schizophrenia spectrum disorder, (*n* = 20).

Patients with SSD, compared to HC, exhibited largely comparable temporal dynamics of BOLD responses in clusters primarily comprising the bilateral precentral gyrus, bilateral putamen, right fusiform gyrus, right middle temporal gyrus, right supramarginal gyrus, left supplementary motor area, left cuneus, and lobule VI of left cerebellar hemisphere as shown in (Table 2, Fig. 2c).

#### Group differences in the temporal dynamics of BOLD responses (active>passive)

We analysed whether patients with SSD differed in the active (compared to passive) movement specific earlier activation patterns observed in HC by applying inclusive masking to the group interaction with the HC contrast. Compared to HC, SSD patients exhibited delayed BOLD activation patterns in several key clusters mainly consisting the right caudate nucleus, lobule VIII of the right cerebellar hemisphere, left superior temporal gyrus, left postcentral gyrus, left thalamus, and left putamen during active compared to passive hand movements (Table 2, Fig. 2d).

#### Interaction analysis

A three-factor interaction of group (HC > SSD), movement condition (active > passive), and video feedback (own hand > other hand) revealed clusters comprising mainly bilateral insula, left postcentral gyrus, and left superior frontal gyrus (Table 2, Fig. 2e). In contrast to HC, SSD patients demonstrated relatively earlier activation in the left precentral gyrus, left supplementary motor area, and left postcentral gyrus during active relative to passive movements with their own hand feedback (Table 2).

#### Group timing differences during active movements with own-hand feedback

We further investigated whether specific (sub-) cortical regions exhibited timing differences during active movement with own hand feedback. Interestingly, HC showed earlier activation than SSD patients for active vs passive movements with own hand feedback, within both hemispheres the clusters primarily comprising the bilateral putamen and bilateral insula (Table 2, Fig. 2f).

#### Group differences in temporal dynamics of BOLD responses (passive > active)

Although contrast (passive > active) revealed distinct clusters within the group of HC and SSD patients, no commonalities were found with similarly activated timing of BOLD responses. Also, no group differences were found. Thus, group differences in earlier activation patterns are specific to active compared to passive movement. However, group differences driven by interaction revealed clusters mainly comprising the left paracentral lobule, left middle temporal gyrus, lobule VIII of left cerebellum, and right parahippocampal gyrus, where HC exhibited relatively earlier responses than SSD during passive movement with own hand feedback (Table 3).

#### Duration of BOLD activation (active > passive)

**Fig. 3:**
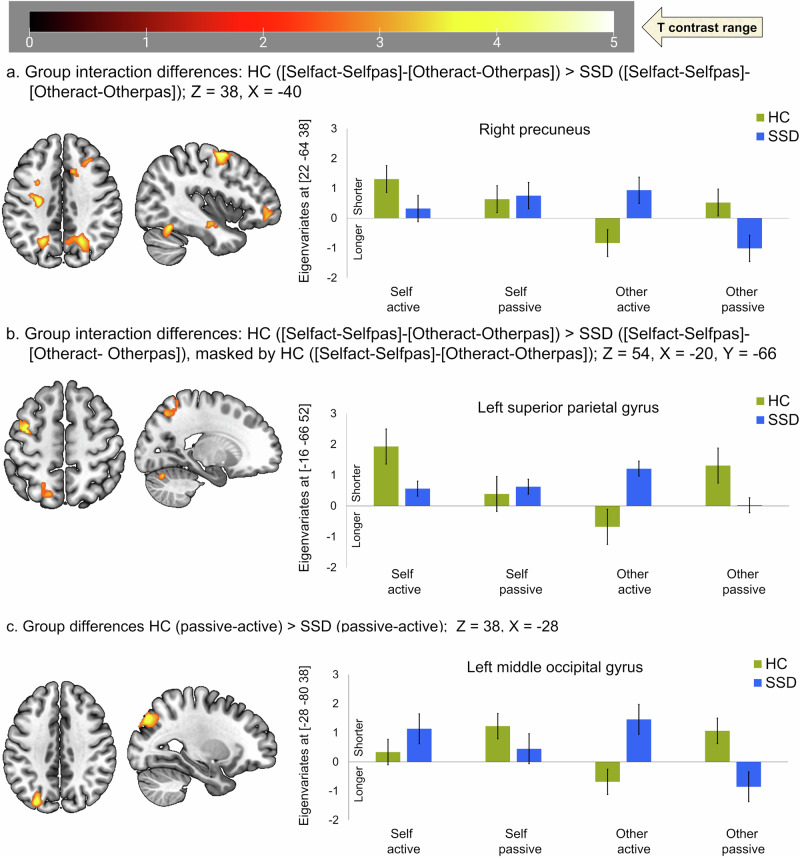
Duration of neural activation in active compared to passive and vice versa. **a** Group interaction: group (HC > SSD) X movement (active>passive) X feedback (own>other) hand shown at Z = 38, X = −40. **b** Group interaction masked by HC X movement (active>passive) X feedback (own>other) shown at Z = 54, X = −20. **c** Group differences between passive and active movement regardless of feedback condition are shown at Z = 38, X = −28. Positive eigenvariates reflect shorter activation durations, while negative ones reflect longer activation durations in the bar graph. HC healthy control, (*n* = 20); SSD schizophrenia spectrum disorder, (*n* = 20).

The conjunction analyses revealed no overlap in activation for the active > passive contrast regarding durations (DD) between HC and SSD patients. Main interaction of group (HC > SSD), movement condition (active > passive), and feedback (own hand > other hand) analyses revealed clusters comprising primarily the left precentral gyrus, right caudate nucleus, left superior parietal gyrus, right middle temporal gyrus, right precuneus, right parahippocampal gyrus, and left middle frontal gyrus (Table 4, Fig. 3a). Additionally, to explore these differences in areas showing a specific pattern in HC, the interaction of group (HC > SSD), movement condition (active > passive), and feedback (own hand > other hand) were masked by either the interaction in HC (movement condition [active > passive] and feedback [own hand > other hand]) or masked by active movement with own hand feedback in HC. Both of these analyses revealed clusters mainly consisting left precentral gyrus, right caudate nucleus, left superior parietal gyrus, and lobule VI of left cerebellar hemisphere (Table 4, Fig. 3b).

#### Duration of BOLD activation/suppression (passive>active)

The (passive > active) contrast did not reveal any regions with similar durations, nor any cluster for the interaction of group (HC > SSD), movement condition (passive > active), and video feedback (own hand > other hand). However, group differences (HC > SSD) with movement condition (passive > active) and video feedback of (own>other) hand revealed clusters comprising mainly the left middle occipital gyrus (Table 4, Fig. 3c).

#### Exploratory correlation analyses

We explored the correlation between temporal dynamics of BOLD responses reflected in TD. Specifically, sub-cortical areas with earlier activation during active movement with own hand feedback (bilateral insula and putamen), and all positive and negative symptom scores were negatively related. A negative correlation indicates, that earlier processing (high or positive value for TD) is related to fewer symptoms, or the other way around: later processing (small or negative value for TD) is related to more symptoms. The timing of the left insula and putamen were negatively correlated to delusions of reference, delusions of being controlled, delusions, bizarre behaviour, residual positive symptoms, affective flattening or blunting, alogia, avolition/apathy, anhedonia/asociality, and total SANS score. The timing of the right insula and putamen was negatively correlated to delusions of reference, delusions of being controlled, delusions, residual positive symptoms, affective flattening or blunting, alogia, avolition/apathy, anhedonia/asociality, and total SANS score (Supplementary Table 1). However, partial correlation analyses controlling for total SANS score revealed no significant correlation (see Supplementary Table 2).

To assess the robustness of observed correlations, Spearman’s rank correlations were further computed and reported in Supplementary Tables 1 and 2. These non-parametric correlations did not reach statistical significance, suggesting that the original Pearson correlations may have been influenced by extreme values or underlying non-linear associations. Furthermore, when applying correction for multiple comparisons, none of the correlations remained significant, underscoring the need for cautious interpretation of these findings.

In a few HCs, we observed between a questionable to mild level of positive and negative symptom scores. However, included HCs did not have any history of neurological or psychiatric conditions, nor a family member with schizophrenia like disorders. Several review paper reported that these mild symptoms may resemble the psychotic like experiences (e.g., hallucinations/delusions) reported in healthy subjects, a transdiagnostic phenomenon common in HC, but transient and occurs at some point in life due to a wide range neurological and psychological circumstances, but not recurring^72–75^.

## Discussion

In this study, we investigated the temporal dynamics of BOLD responses during video feedback from active and passive hand movements in patients with schizophrenia spectrum disorders (SSD). To our knowledge, this is the first fMRI study to explore such fine-grained timing differences using the temporal derivative (TD) and dispersion derivative (DD) of the hemodynamic response function (HRF). Our results demonstrate that patients with SSD exhibit delayed neural activation for feedback of active movements compared to HC in the right caudate nucleus, lobule VIII of the right cerebellum, left superior temporal gyrus, left postcentral gyrus, left thalamus, and left putamen and insula. These regions are critically involved in feedback monitoring and agency attribution, while their delayed activation may reflect aberrant CD and EC functioning and cortical-subcortical desynchronisation—the potential core mechanisms underlying impaired sensory-motor integration and sense of agency in SSD.

This study has shown similar BOLD response timing in patients with SSD and HC in the bilateral precentral gyrus, bilateral putamen, right fusiform gyrus, right middle temporal gyrus, right supramarginal gyrus, left supplementary motor area, left cuneus, and lobule VI of left cerebellar hemisphere (shown in Table 2, Fig. 2c). However, earlier BOLD responses in active versus passive hand movements may support the notion that typically active hand movement induces earlier neural responses. In this regard our study in HC has inferred that EC-based predictive mechanisms may lead to earlier neural processing of the visual feedback for active hand movement in contrast to passive ones^47^. This interpretation also aligns with EEG studies suggesting that lateralised-/readiness potential (L-/RP) occurs starts before movement^48,49^ manifested in earlier BOLD responses. However, it’s not straightforward distinction, for instance, contrast to HC, SSD has shown relatively earlier activation (active>passive) pattern in the left precentral gyrus, left supplementary motor area, and left postcentral gyrus (Table 2) in the interaction analyses. These area may reflect altered early cognitive volitional control in CD, while the delayed activation in patients with SSD (Table 2, Fig. 2d–f) may stem from imprecise CD mediated delayed and imprecise EC, leading to often reported neuropathology of psychomotor abnormalities, movement intention, delayed/disrupted sensory-motor integration, and giving an imprecise feeling of control (agency)^6,7,9,12,35,36,48,55^. These may indicate that the imbalance in BOLD response timing in SSD patients might be associated with a reduced movement-feedback control stability.

**Fig. 4:**
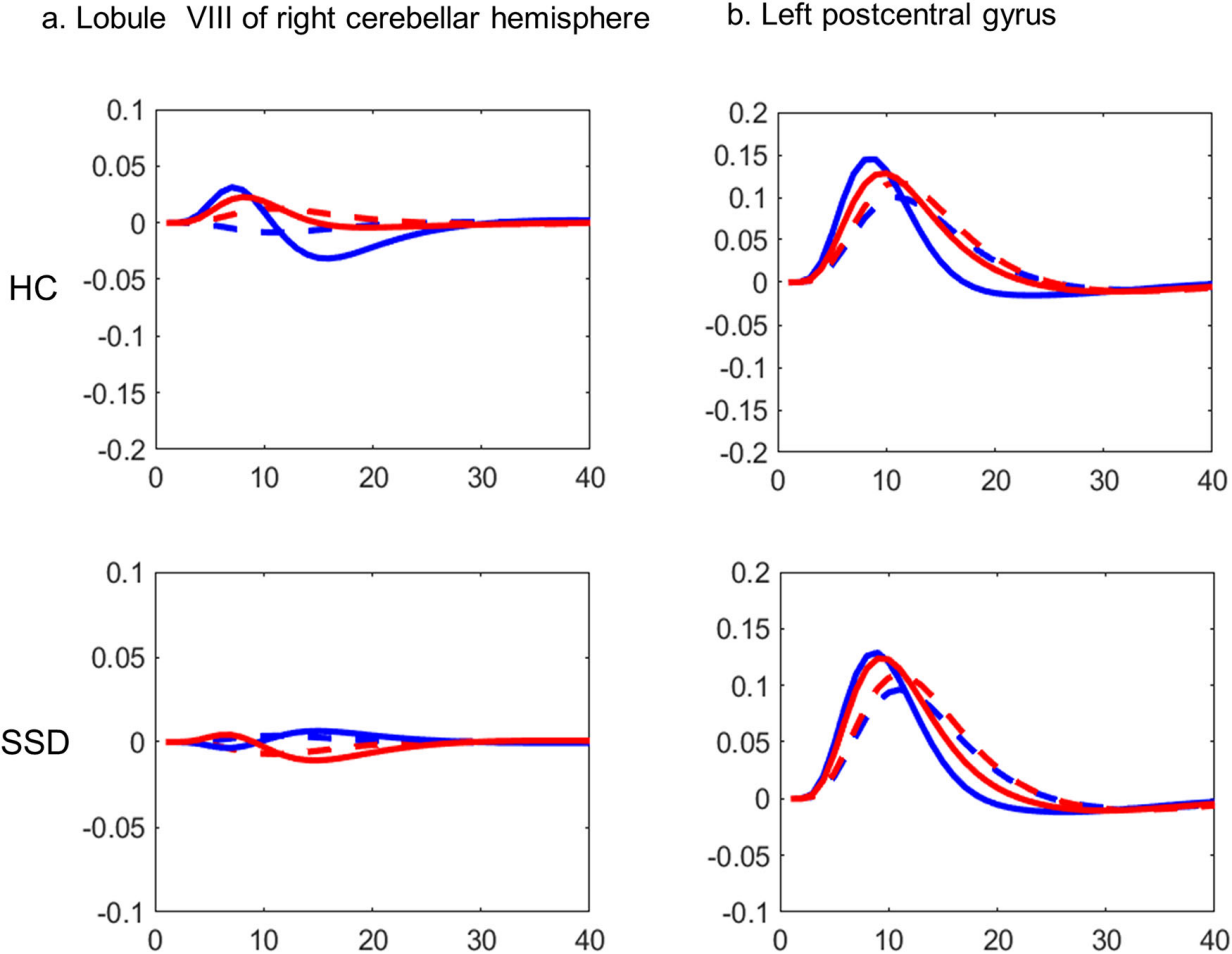
Latency functions (LF)^47,65^ from TD and from the canonical hemodynamic response function (HRF) in healthy control (upper row) and in schizophrenia spectrum disorder (SSD) in the lower row. **a** LF at the lobule VIII of the right cerebellar hemisphere, and (**b**) LF at the left post central gyrus. Active: canonical (dashed blue), HRF with temporal derivative (solid blue); Passive: canonical (dashed red), HRF with temporal derivative (solid red). HC healthy control, (*n* = 20); SSD schizophrenia spectrum disorder, (*n* = 20).

In addition to delayed timing (Table 2, Fig. 2d–f), their atypical latency function (Fig. 4), and altered durations of neural responses (Table 4, Fig. 3a–c) provide a compelling evidence for impaired temporal dynamics in patients with SSD. Which may leading to a disrupted timing and durations of cortical-subcortical synchronisation across the brain, likely contributing to the imprecise differentiation between active and passive movement authorship. While direct evidence is limited, movement abnormalities, excitation-inhibition imbalance, and psychomotor abnormalities may explain our altered temporal dynamics of BOLD responses in SSD patients^12,36^. Furthermore, delayed responses in lobule VIII of the right cerebellum, left superior temporal gyrus, left postcentral gyrus, left thalamus, and left putamen and insula might support the notion of desynchronised neural responses across cortico-ponto-thalamo-cerebellar circuits; which may disrupt the transformation of motor intention into sensory-motor predictions and error corrections during sensory-motor feedback integration and monitoring. This is supported by fMRI studies in SSD patients, which have shown deficits in these pathways, including aberrant connectivity between the thalamus, pons, and cerebellum^42–45^. Similarly, a review paper supports that psychomotor mediated neural activity patterns are abnormally balanced and dynamic synchronisation across networks and brain regions is aberrant in schizophrenia^76^. Further studies demonstrated that temporal variability of functional connectivity across time is disrupted in early visual areas, the thalamus, and the temporal cortex in patients with SSD^77^. Another study reported abnormal dynamic changes in brain activity across time in schizophrenia^78^. Thus, our findings may reflect a diminished dynamic ability to process awareness of intended hand movement and feedback (agency) monitoring in patients with SSD.

Specific delayed activation effect during active hand-movement with own-hand feedback observed in the bilateral insula (Table 2, Fig. 2f), which is extended to putamen. In patients with SSD, delayed responses in the bilateral insula-putamen were associated with higher symptom severity (Supplementary Table 1), although this association was attenuated after controlling for negative symptom load (Supplementary Table 2). Importantly, these exploratory correlations did not survive correction for multiple comparisons, especially because we performed a large number of correlations. However, attenuated when applying Spearman’s Rank correlation, most likely due to smaller sample size, moderate effect sizes, or non-linear model assumptions. These findings may reflect exploratory trends or general symptom effects rather than symptom-specific mechanisms. However, the insula is often reported as impaired in SSD, which is the core hub of the self-processing and salience network, linked to the feeling of causal-initiator of intended action, synchronisation of internal-external sensory information, emotional context of action awareness, and prediction-error resolution in feedback monitoring^2,34,55,57,79–88^. All these processes can be linked to several positive and negative symptoms, ranging from ego-disturbances to asociality or avolition. Damage in the insula was implicated in disturbed self-action awareness in patients across psychiatric^20,57,88^, neurological^20,89^, and neurodegenerative disorders^89–91^. In addition to left insula/putamen, areas found with reduced preparatory BOLD amplitude^56^ has also shown delayed response during active hand-movement with own hand feedback, particularly in the bilateral insula/putamen, left thalamus, and lobule VIII of right cerebellum (Table 2, Fig. 2d–f) and atypical latency function (Fig. 4)). Which may collectively reflect disrupted underlying neurophysiological mechanisms of often reported disrupted coordination across the brain and the impaired sense of agency in patients with SSD^42–45,92^. Furthermore, given the strong overlap in correlation patterns with delayed responses from bilateral insula/putamen, these findings highlights the role of bilateral insular dysfunction contributing to impaired sensorimotor integration, self-referential processing, and sense of agency in SSD. Thus, one of the potential underlying translational target mechanisms to improve the neural synchronisation, sense of agency, and ego-disturbances.

Regarding the duration of BOLD responses, patients with SSD demonstrated relatively longer processing for active movement with own hand feedback in the left precentral gyrus, right caudate nucleus, left superior parietal gyrus, and lobule VI of left cerebellar hemisphere (Table 4, Fig. 3b). Although most distinct in active movement with own hand feedback, it is noticeable that durations of neural processing are altered in these brain areas in patients with SSD. Furthermore, regardless of the feedback conditions in the (passive > active) contrast, in contrast to HC, patients with SSD demonstrated longer processing for passive but shorter for active movements in the left middle occipital gyrus (Table 4, Fig. 3c). Nevertheless, direct evidence regarding duration abnormalities are scarce, these first evidence of altered patterns of durations in these areas are mostly related to sensory-motor integration and monitoring active-passive movement authorship, especially with own hand feedback^17^. Although not a direct measurement of psychomotor slowing, significant difference in our trail making task (TMT B-A; see in Table 1) may reflect reduced executive-psychomotor functioning and visuo-motor flexibility in patients with SSD. This aligns with consistent reports of psychomotor abnormalities, impaired sensory-motor feedback integration^10–14^ and action-consequences monitoring^10,11,15–20^ in patients with SSD.

Our findings largely align with the previous evidence in HC^47^ showing earlier activation during active compared to passive hand movements. Importantly, our findings further demonstrate aberrant timing patterns in SSD patients (Table 2, Fig. 2d–e). Regarding durations of BOLD responses, shorter durations are more specific to active compared to passive movements, particularly with own hand feedback in HC, but this distinction was altered in SSD patients. These findings align with previous findings that patients with SSD exhibit impairments in BOLD response amplitudes^3,56^, while disrupted neural responses’ timing and duration of hand movement might indicate that formation and functions of CD and EC are impaired, which may additionally underlie temporally imprecise integration of movement intentions with sensory feedback and likely facilitates key symptoms in SSD. These interpretations are supported by EEG studies of finger and brisk right fist closure movement in SSD patients, which demonstrated abnormal/altered action awareness manifested with L-/RP amplitudes (reduced, delayed, mismatched in durations) typically emerge approximately 2 s earlier than movement; along with deficits in post-movement event-related synchronisation^37,93–95^. These abnormalities in temporal dynamics may reflect the sensorimotor integration abnormalities, potentially contributing to self-disorder in schizophrenia^94^. Taken together, our findings emphasise the importance of considering the temporal dynamics of neural processing when examining sensory-motor dysfunctions in schizophrenia, which are undetectable by canonical analyses of BOLD amplitudes^3^. By mapping and understanding these abnormalities in the temporal dynamics of BOLD responses, future studies could extend these methods in large samples with sub-symptom groups. Three-dimensional (amplitude, timing, and duration) assessment of abnormalities might facilitate defining the neural mechanisms’ characteristics of sensory-motor dysfunction and self-disorder in patients with SSD.

## Limitations

In general our study has shown abnormalities in the BOLD response timing and duration in patients with SSD. However, we would like to address few limitation that could be considered in future studies. First, the relatively small sample size, while common in fMRI studies due to limited patients available, particularly in the correlation analyses. The significance strength with Spearman’s rank correlations were weakened, while the application of statistical thresholds like family-wise error correction and correction for multiple comparisons has led to the disappearance of significance. Second, although our patient sample was diagnostically homogeneous, it was slightly heterogeneous with respect to pharmacological treatment. The majority of patients (*n* = 15) were taking second-generation antipsychotics (SGAs), one was on a first-generation antipsychotic (FGA), and the remaining four were not using antipsychotics but other medications. However, it has been widely reported that motor and agency abnormalities are exist in patients with psychosis, first-episode schizophrenia, and drug naïve schizophrenia too, and even in other neurological and neurodegenerative disorders with no antipsychotics use history^12,96,97^. Consistent with this, we also found no correlation between neural response timing in the insula/putamen and olanzapine-equivalent dosage in our sample. Thus, while a minor impact of antipsychotic medication cannot be entirely ruled out, our findings on BOLD response timing and duration abnormalities appear robust and extend beyond this limitation. Third, the basis function modelling used in this study introduces its own trade-offs. While the TD improves sensitivity to timing shifts and the DD captures differences in response duration, both can reduce sensitivity to amplitude changes^66,67^. However, our sequential use of HRF + TD and HRF + TD + DD models was the best fit to balance these sensitivities, still, some inevitable subtle amplitude-specific effects may have gone undetected depending on model choice. These complementary strengths and trade-offs should be considered when interpreting results across models incorporating TD and DD. In future experiments, large samples, potentially with sub-symptom groups, would be encouraged to map timing and durations in the whole brain. Furthermore, future studies could separate and investigate the timing for preparation and execution of hand movement, and how patients with SSD differ relative to HC. This could be an extension of previous findings of impaired preparatory neural activation patterns identified in patients with SSD^56^.

## Conclusion

In this fMRI study, we investigated BOLD responses’ timing using TD and duration using DD during the processing of active vs. passive hand movements with own or other hand video feedback. For the first time with fMRI study, we revealed commonalities and differences in BOLD responses’ timing and durations between HC and patients with SSD. Our findings demonstrate aberrant temporal dynamics of BOLD responses in patients with SSD, characterised by delayed and deviating BOLD responses’ duration during active compared to passive hand movements. Our results suggest that these BOLD responses’ characteristics may reflect deficits in the early predictive mechanisms and hand-movement feedback monitoring of schizophrenia. The distinct timing and duration pattern of the BOLD responses’ across cortical and subcortical regions may indicate that temporal synchronization between areas is impaired, most likely due to imprecise or delayed corollary discharge and efference copy mechanisms. Such disruptions affect the temporal integration of internal and external signals, compromising the coordination needed for movement control and authorship. Notably, the reduced differentiation between active and passive movement-related BOLD responses in the bilateral insula-putamen and lobule VIII of the right cerebellar hemisphere, alongside the negative correlation between delayed BOLD responses in the insula-putamen and symptom severity, further highlight the link between these neural disruptions and disturbances in self-awareness and agency in schizophrenia. This study underscores the critical role of BOLD responses’ timing and duration in understanding sensory-motor dysfunction in schizophrenia, offering new insights into the neural mechanisms characteristics underlying impaired action awareness, motor dysfunctions, and agency perception.

## Supplementary information

Revised Supplementary Material

## Data Availability

The data information supporting this study’s findings is published in the following repository link: 10.5281/zenodo.15806068.
